# Exhaled CO_2_ Parameters as a Tool to Assess Ventilation-Perfusion Mismatching during Neonatal Resuscitation in a Swine Model of Neonatal Asphyxia

**DOI:** 10.1371/journal.pone.0146524

**Published:** 2016-01-14

**Authors:** Elliott Shang-shun Li, Po-Yin Cheung, Megan O'Reilly, Joseph LaBossiere, Tze-Fun Lee, Shaun Cowan, David L. Bigam, Georg Marcus Schmölzer

**Affiliations:** 1 Faculty of Science, McGill University, Montreal, Quebec, Canada; 2 Department of Pediatrics, University of Alberta, Edmonton, Alberta, Canada; 3 Centre for the Studies of Asphyxia and Resuscitation, Neonatal Research Unit, Royal Alexandra Hospital, Edmonton, Alberta, Canada; 4 Department of Surgery, University of Alberta, Edmonton, Alberta, Canada; 5 Faculty of Science, University of Alberta, Edmonton, Alberta, Canada; Icahn School of Medicine at Mount Sinai, ARGENTINA

## Abstract

**Background:**

End-tidal CO_2_ (ETCO_2_), partial pressure of exhaled CO_2_ (PECO_2_), and volume of expired CO_2_ (VCO_2_) can be continuously monitored non-invasively to reflect pulmonary ventilation and perfusion status. Although ETCO_2_ ≥14mmHg has been shown to be associated with return of an adequate heart rate in neonatal resuscitation and quantifying the PECO_2_ has the potential to serve as an indicator of resuscitation quality, there is little information regarding capnometric measurement of PECO_2_ and ETCO_2_ in detecting return of spontaneous circulation (ROSC) and survivability in asphyxiated neonates receiving cardiopulmonary resuscitation (CPR).

**Methods:**

Seventeen newborn piglets were anesthetized, intubated, instrumented, and exposed to 45-minute normocapnic hypoxia followed by apnea to induce asphyxia. Protocolized resuscitation was initiated when heart rate decreased to 25% of baseline. Respiratory and hemodynamic parameters including ETCO_2_, PECO_2_, VCO_2_, heart rate, cardiac output, and carotid artery flow were continuously measured and analyzed.

**Results:**

There were no differences in respiratory and hemodynamic parameters between surviving and non-surviving piglets prior to CPR. Surviving piglets had significantly higher ETCO_2_, PECO_2_, VCO_2_, cardiac index, and carotid artery flow values during CPR compared to non-surviving piglets.

**Conclusion:**

Surviving piglets had significantly better respiratory and hemodynamic parameters during resuscitation compared to non-surviving piglets. In addition to optimizing resuscitation efforts, capnometry can assist by predicting outcomes of newborns requiring chest compressions.

## Introduction

Neonatal asphyxia is a common cause of mortality and morbidity and worldwide contributes to approximately 1 million deaths annually. It has been reported that 0.08% of term-born neonates required cardiopulmonary resuscitation (CPR) [1]. In the latest guidelines on neonatal resuscitation, the American Heart Association states that if the heart rate remains undetected after 10 minutes in asystolic neonates, discontinuing the resuscitation efforts is justified [2]. However, the decision to discontinue resuscitation may be influenced by issues such as the presumed aetiology of the arrest, gestation of the baby, potential reversibility of the situation, and parents’ previously expressed feelings about the acceptable risk of morbidity. Thus, an objective method to assess recovery or to predict success of resuscitation may help decision-making.

End-tidal CO_2_ (ETCO_2_) is the level of CO_2_ at the end of an exhaled breath and is mainly determined by alveolar ventilation, pulmonary perfusion (right ventricular output), and total body CO_2_ production due to metabolism [3]. During acutely low cardiac output states, such as cardiac arrest, decreased pulmonary flow becomes the primary determinant of ETCO_2_, resulting in low ETCO_2_ values [4,5]. Observing changing levels of ETCO_2_, which reflect changes in pulmonary blood flow, while delivering chest compressions (CC) and ventilations has proven to be useful in determining circulatory status during cardiac arrest and resuscitation in human adults [6]. In theory, if all steps of CPR are performed adequately (e.g. delivering adequate ventilation (breathing), and performing CC (circulation)), ETCO_2_ values should be normal. Thus, low ETCO_2_ could be an indicator of ineffective CC and/or ventilation, which potentially could be used to improve the efficacy of the resuscitation in real time.

Partial pressure of exhaled CO_2_ (PECO_2_), which can be monitored non-invasively, reflects ventilation-perfusion matching. Other methods commonly used to measure ventilation-perfusion matching involve invasive or isotopic techniques that are not feasible in the neonatal population. To our knowledge, no studies regarding monitoring PECO_2_ have been done during CPR or within the neonatal population.

Using a swine model of neonatal hypoxia and asphyxia, we aimed to examine the temporal changes of ETCO_2_, volume of expired CO_2_ (VCO_2_), PECO_2_ and their relationship with survivability and hemodynamic changes during CPR. Based on the principle of animal experimentation, we reviewed the data collected in our previous experiments [7,8] in an attempt to discover an objective approach to evaluate recovery or predict the outcome of resuscitation. We hypothesized that ETCO_2_ and PECO_2_ during CPR correlated with hemodynamic changes and preceded the return of spontaneous circulation (ROSC) in asphyxiated newborn piglets.

## Methods

**Fig 1:**
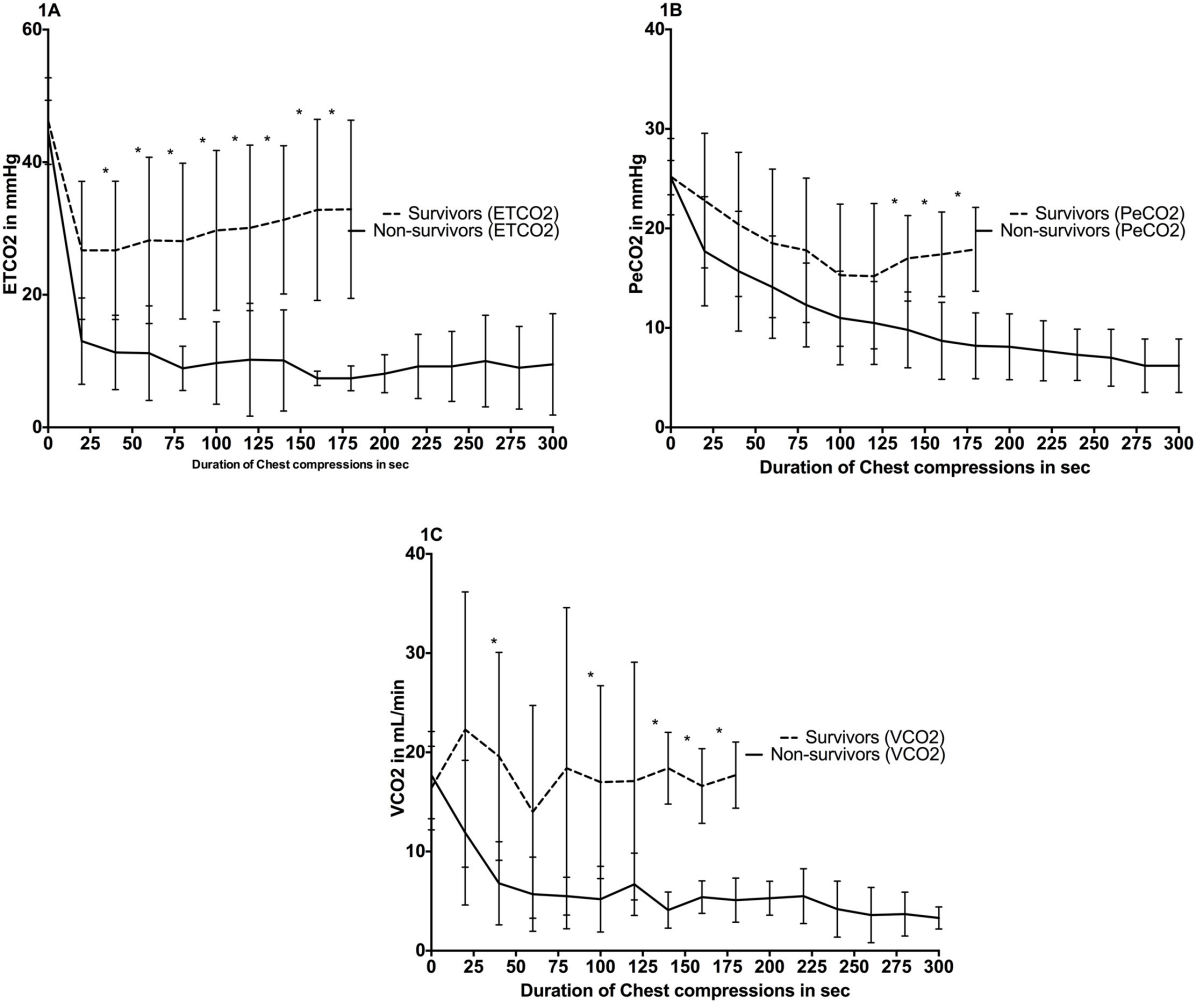
Study protocol.

Respiratory data was recorded for twenty newborn mixed breed piglets (1–4 days of age, weighing 1.6–2.3 kg), which were obtained on the day of experimentation from the University Swine Research Technology Centre. All experiments were conducted in accordance with the guidelines and approval of the Animal Care and Use Committee (Health Sciences), University of Alberta and presented according to the ARRIVE guidelines [9]. The piglets were instrumented as previously described [7,8]. A graphical display of the study protocol is presented in Fig 1. Animal ethics protocol number: AUP00000237.

### Respiratory parameters

A respiratory function monitor (NM3, Respironics, Philips, Andover, MA) was used to continuously measure tidal volume (V_T_), airway pressures, gas flow, ETCO_2_, VCO_2_, and PECO_2_. The combined gas flow and ETCO_2_ pneumotachometer was placed between the endotracheal tube and the ventilation device. Gas flow and airway pressures were measured using a fixed orifice flow pneumotachometer. V_T_ was calculated by integrating the flow signal. ETCO_2_ was measured with a mainstream sensor using non-dispersive infrared absorption.

### Experimental protocol

We reviewed data from our previous experiments [7,8], where piglets were randomized to receive either coordinated CPR with 3:1 compression:ventilation ratio vs. continuous CC during sustained inflations (SI) [7] or 3:1 compression:ventilation ratio vs. continuous CC with non-synchronized ventilation (CCaV) [8]. Briefly, all piglets were exposed to 45 minutes of normocapnic hypoxia. Hypoxia was followed by asphyxia until heart rate decreased to 25% of baseline, which was achieved by disconnecting the ventilator and clamping the endotracheal tube. Fifteen seconds after heart rate reached 25% of baseline, positive pressure ventilation was conducted for 30 seconds prior to the initiation of CC. ROSC was defined as an increase in heart rate >150/min for 15 seconds (Fig 1).

Technique of Resuscitation: Positive pressure ventilation was provided with a Neopuff T-Piece (Fisher & Paykel, Auckland, New Zealand); default settings were a peak inflating pressure of 30 cmH_2_O, a positive end expiratory pressure of 5 cmH_2_O, and a gas flow of 8 L/min. CC were performed using the two-thumb encircling technique by a single operator (GMS) in all piglets. A metronome was used to achieve the targeted CC rate. After 30 seconds of CC, oxygen was increased from 21% to 100%. Epinephrine was administered if no increase in heart rate or ROSC was observed despite adequate ventilation and CC. One minute after CC were commenced, epinephrine (0.01 mg/kg per dose) was given intravenously and then every minute as needed to a maximum of four doses. CPR in the 3:1 group was performed according to the current resuscitation guidelines with 90 CC and 30 inflations per minute [2]. Piglets randomized to the SI group received a SI with a peak inflating pressure of 30 cmH_2_O for 30 seconds. During SI, CC with a rate of 120 per minute was provided. SI was interrupted after 30 seconds for one second before a further 30 seconds of SI was provided. CC was delivered continuously until ROSC was achieved. Piglets randomized to CCaV received continuous CC at a rate of 90 CC/minute and asynchronous ventilations at a rate of 30 ventilations/minute. After ROSC, piglets were allowed to recover for four hours, and were then euthanized with an intravenous overdose of phenobarbital (100 mg/kg).

### Data collection and analysis

Demographics of study piglets were recorded. Transonic flow probes, heart rate, and pressure transducer outputs were digitized and recorded with custom Asyst programming software (Data Translation, Ontario, Canada). Peak inflating pressure, V_T_, ETCO_2_, PECO_2_, and VCO_2_ were measured and analyzed using Flow Tool Physiologic Waveform Viewer (Philips Healthcare, Wallingford, CT). Respiratory function data were available for a total of 17 asphyxiated piglets, due to a malfunction of the respiratory function monitor [7,8]. Cardiac index (CI) was calculated using pulmonary artery blood flow (PABF)/body weight. The maximum duration of CC was 300 seconds; for analysis, we grouped the data into 5-second epochs. The data are presented as mean ± standard deviation (SD) for normally distributed continuous variables and median (interquartile range—IQR) when distribution was skewed. For all respiratory parameters, continuous values during CPR were analyzed. The data was tested for normality and compared using Student’s *t-test* for parametric and the Mann-Whitney *U*-test for nonparametric comparisons of continuous variables; χ^2^ was used for categorical variables. *P*-values are 2-sided and p<0.05 was considered statistically significant. Statistical analyses were performed with Stata (Intercooled 10, Statacorp Tx).

## Results

Seventeen piglets were exposed to normocapnic hypoxia and asphyxia prior to receiving CPR. Baseline characteristics are presented in Table 1. The heart rate prior to commencement of CPR was similar between survivors and non-survivors (Table 1). The period of asphyxia was also similar between survivors and non-survivors (Table 1).

**Table 1:** Characteristics at baseline and prior to commencement of Cardio-Pulmonary Resuscitation (CPR).

	Survivors (n = 10)	Non-survivors (n = 7)	p-value
**Baseline characteristics**			
Age (days)	2 (1)	3(1)	0.10
Weight (g)	1830 (141)	1800 (141)	0.67
Male/female	7/2	7	0.21
Heart rate (bpm)	229 (22)	252 (26)	0.08
Arterial pH	7.37 (0.05)	7.33 (0.05)	0.25
Arterial P_CO2_ (mm Hg)	45 (4)	47 (3)	0.40
Plasma lactate (mmol/L)	3.9 (0.6)	4.2 (1.3)	0.46
Arterial hemoglobin (g/L)	83 (10)	80 (12)	0.54
**Characteristics at commencement of CPR**			
Asphyxia time (sec)^#^	87 (55–120)	102 (72–135)	0.26
Heart rate prior CPR (bpm)^#^	33 (0–49)	58 (38–63)	0.13
Arterial pH	6.96 (0.1)	6.89 (0.1)	0.21
Arterial P_CO2_ (mm Hg)	79 (20)	84 (24)	0.65
Plasma lactate (mmol/L)	11 (4)	12 (4)	0.87

Data presented as mean (SD) unless indicated ^#^median (IQR)

### Resuscitation

Overall, nine piglets achieved ROSC compared to eight who did not. Oxygen use was similar between groups (Table 2). Swine in both groups required epinephrine; however, the number of administered doses of epinephrine was significantly higher in the non-survivor group compared to the survivor group (Table 2).

**Table 2:** Characteristics during Cardio-Pulmonary Resuscitation (CPR).

	Survivors (n = 10)	Non-survivors (n = 7)	p-value
Oxygen use	8/2	6/1	0.76
Epinephrine use	5/5	6/1	0.13
Doses of epinephrine^+^	1 (0–4)	3 (0–4)	0.042
Mean arterial pressure (mm Hg)	40 (16)	34 (4)	0.25
Pulmonary arterial pressure (mm Hg)	36 (11)	30 (6)	0.13
Central venous pressure (mm Hg)	26 (9)	26 (9)	0.99

Data presented as mean (SD) unless indicated ^+^mean (range)

### Respiratory parameters

**Fig 2:**
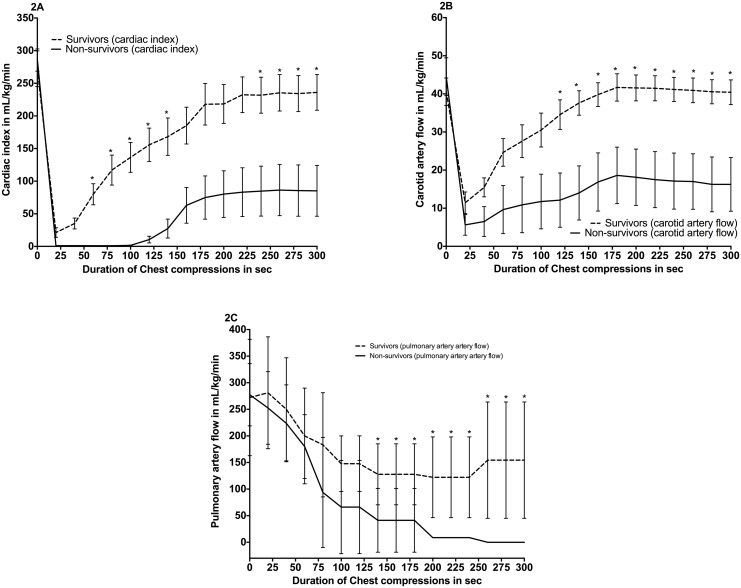
ETCO_2_ (1A), PeCO_2_ (1B), VCO_2_ (1C) for survivors vs. non-survivors. Baseline (at “0”, PPV until “30sec”, and CPR thereafter). Data are presented in mean (middle of line) with standard deviation (error bars), (* indicates p<0.05 survivors vs. non-survivors).

ETCO_2_ values were significantly higher in survivors compared to non-survivors during CPR (Fig 2A). PECO_2_ values were also significantly higher in survivors compared to non-survivors after 32 seconds of CC (Fig 2B). VCO_2_ values were observed to be significantly higher in survivors compared to non-survivors during CPR (Fig 2C).

Median (IQR) V_T_ delivery and peak inflation pressure were similar between survivors and non-survivors; 14.8 (12.7–18.7) mL/kg and 30 (29–31) cmH_2_O compared to 14.5 (12.5–17.8) mL/kg, and 30 (30–30) cmH_2_O, respectively.

### Hemodynamic parameters

**Fig 3:**
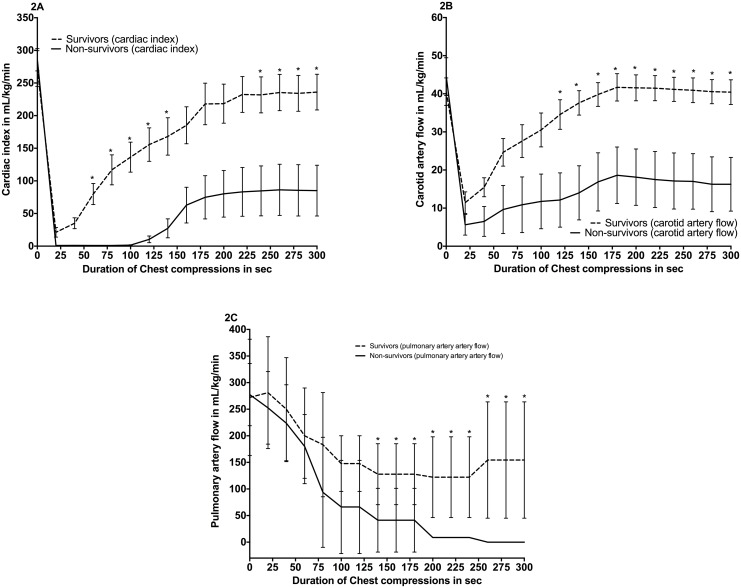
Cardiac index (2A), carotid artery flow (2B), pulmonary artery flow (2C) for survivors vs. non-survivors. Baseline (at “0”, PPV until “30sec”, and CPR thereafter). Data are presented in mean (middle of line) with standard deviation (error bars), (* indicates p<0.05 survivors vs. non-survivors).

Piglets in the survivor group had a significantly increased median (IQR) carotid artery blood flow (CABF) from 120 seconds of CC onwards and significantly increased CI for the entire duration of CC compared to non-survivors (Fig 3A–3C). PABF was significantly increased in the latter part of resuscitation between survivors and non-survivors (Fig 3C). No differences between groups regarding mean arterial blood pressure, pulmonary artery blood pressure, and central venous pressure were observed (Table 2).

## Discussion

In this study, we analyzed data from our experiments using an established swine model of neonatal asphyxia and resuscitation [10] to assess hemodynamic and respiratory parameters, which could potentially provide a clinical indicator to achieve ROSC. Our results indicated that surviving piglets had significantly higher values of ETCO_2,_ VCO_2_, and PECO_2_ values during CPR compared to non-surviving piglets (Fig 2A–2C). In addition, we observed significantly higher CABF and CI during CPR in surviving piglets (Fig 3A and 3B). Surviving piglets also required significantly less epinephrine administration. Our data suggests that continuously monitoring ETCO_2_, VCO_2_, and PECO_2_ during CC has the potential to be a non-invasive measurement to indicate ROSC, support prediction of outcomes in newborns requiring CC, and determine whether resuscitation efforts could be discontinued.

In the adult population, changes in ETCO_2_ are used to evaluate ventilation and cardiac output during situations such as CPR [6]. A study using the same porcine model of hypoxia and asphyxia reported that even in low cardiac output states, positive CO_2_-detector results are observed [10]. In addition, ETCO_2_ has been detected during bradycardia (heart rate <50 beats/min) in an extremely preterm newborn [11]. Observing changing levels of ETCO_2_, which reflect changes in pulmonary blood flow, while delivering CC and ventilations has proven to be useful in determining circulatory status during cardiac arrest and resuscitation in human adults [6]. Furthermore, ETCO_2_ is good indicators of adequate establishment of the three components of CPR: airway, breathing, and circulation. In our study, piglets that achieved ROSC had significantly higher ETCO_2_ levels throughout the duration of CPR compared to piglets that did not achieve ROSC (Fig 2A). *Chalak et al* reported similar results by predicting ROSC using capnometry in a neonatal porcine model [3]. Using an ETCO_2_ of 14 mmHg was the most reliable indicator for ROSC with 92% sensitivity and 81% specificity. *Chalak et al* suggested that monitoring ETCO_2_ trends during resuscitation would allow uninterrupted CC and could provide a better indicator of the effectiveness of perfusion during CC [3]. This hypothesis was confirmed in an extremely preterm newborn where an increase in ETCO_2_ preceded and successfully predicted ROSC [11]. ETCO_2_ monitoring is a non-invasive tool that has been shown to predict and demonstrate ROSC during both animal and human adult cardiac arrest [6, 12–15]. Guidelines currently do not have any recommendations for the use of qualitative colorimetric CO_2_-detector during CPR; however, it may be useful to implement qualitative colorimetric CO_2_-detector or quantitative detectors measuring ETCO_2_, VCO_2_, and PeCO_2_ as a means to guide resuscitation efforts during CPR, and also serve as a predictor for ROSC.

VCO_2_, or the volume of expired CO_2_, reflects changes in both ventilation and perfusion, and therefore V/Q matching. *Palme-Kilander et al* reported that low VCO_2_ values recorded in preterm infants could originate from a number of factors, including deficient aeration due to residual lung fluid, very low tone, and deficient perfusion of the lungs [16]. Furthermore, a recent study showed that higher VCO_2_ levels are associated with lung aeration and successful establishment of a functional residual capacity [17]. Survivors in our study had significantly higher VCO_2_ during some portions of CPR (Fig 2C). Increased levels of VCO_2_ in the survivors reflected adequate ventilation, perfusion, and lung aeration. Thus, VCO_2_ has the potential to be a useful respiratory parameter that provides valuable information during neonatal resuscitation.

PECO_2_ is the partial pressure of exhaled CO_2_ and is a continuous, non-invasive measurement. Since the physiological dead space/tidal volume (VD/VT) ratio is never zero [18], PECO_2_ is always lower than the ETCO_2_ [19]. With poor ventilation to perfusion matching, VD/VT increases, regardless of whether mismatching is due to uneven perfusion, uneven ventilation, or a mixture of uneven perfusion and uneven ventilation, causing a lower PECO_2_. Thus, PECO_2_ is reduced under all conditions of uneven ventilation/perfusion [18]. In the case of ventilation mismatch, PECO_2_ is dilute relative to ETCO_2_, and the PECO_2_/ETCO_2_ ratio is reduced [18]. In the case of reduced or maldistributed pulmonary blood flow without airway defects, both PECO_2_ and ETCO_2_ would be reduced, resulting in a near normal PECO_2_/ETCO_2_ ratio [18]. To our knowledge, no studies regarding monitoring PECO_2_ have been done during CPR or within the neonatal population. Piglets in the current study that successfully achieved ROSC had significantly higher PECO_2_ levels in the latter portion of CPR (Fig 2B), indicating sufficient gas exchange was occurring. Low levels of PECO_2_ can only be attributed to poor or low quality of ventilation during CPR, while depressed levels of both PECO_2_ and ETCO_2_ may signify inadequate pulmonary perfusion due to poor circulation. These findings may have important clinical use; by continuously analyzing PECO_2_ and ETCO_2_ during CPR, resuscitators can determine changes in ventilation or perfusion and adjust ventilation to improve in this context.

In the delivery room pulse oximetry is used to measure oxygen saturation (SpO_2_) to titrate oxygen delivery and monitor heart rate [2]. Pulse oximetry can be used to immediately after birth and, in the majority of cases, heart rate and oxygen saturation are displayed within 90 seconds [19–21]. However, in situations of poor peripheral perfusion (e.g. cardiac arrest or severe bradycardia) reliable signals are not always achieved and therefore relying on pulse oximetry to asses the adequacy of CC can be misleading [18]. Therefore measurements of ETCO_2_, PECO_2_, and VCO_2_ allow resuscitators to assess the quality of their resuscitation and assess changes in ventilation or perfusion [3,22]. Low SpO_2_ values cannot differentiate between poor ventilation and poor perfusion. In addition, no study in newborns has assessed if changes in pulse oximetry waveforms can be used to predict ROSC. In summary, correctly applied pulse oximetry together with capnometry will aid the resuscitator to improve resuscitation performance.

The blood flow at the common carotid artery can serve as a good surrogate of cerebral blood flow in feto-neonatal animals [23]. It has been well documented that cerebral blood flow is related to the arterial partial pressure of CO_2_ (PaCO_2_) [24]. In fact, an increase in cerebral blood flow is caused by an increase in PaCO_2_ [24]. We measured a significantly higher CABF and CI in the surviving group, which demonstrates increased PaCO_2_ and augmented antegrade blood flow just 40 seconds after CC was initiated. This is of clinical importance; by combining this information with capnometry, we can start to assess the quality of CC, attempt to enhance resuscitation efforts to improve ETCO_2_ and PECO_2_ levels, and increase the probability of achieving ROSC within the first minute of resuscitation. If prolonged CPR efforts are given and there is no evidence that ROSC could be attained, it may be appropriate to cease resuscitation after considering all other factors.

## Limitations

All piglets had already undergone fetal to neonatal transition, which limits the applicability to delivery room resuscitation. However, recent studies demonstrated that exhaled CO_2_ could be measured during neonatal transition to guide ventilation [16,22,25]. All piglets were anesthetized and sedated, which differs from delivery room resuscitations. Piglets were intubated using a tightly sealed endotracheal tube to prevent any endotracheal tube leak, which allowed accurate assessment of gas flow for the purpose of this study, but is not a precise replication of the clinical setting where mask ventilation is normally used. In addition, the ductus arteriosus was ligated in all piglets in order to ensure cardiac output could be accurately assessed by PABF. Despite these limitations, the findings are still relevant because the distribution of cardiac output in the fetus and the post-translational neonate during asphyxia episodes are qualitatively similar [26–28]. Piglets were also given different CC techniques, which may have contributed to overall survival and results [7,8]. Since manual ventilations and CC could cause ETCO_2_ to fluctuate with the effort of compression and rate of ventilation [15,29], uniform CC and a constant rate of ventilation needs to be delivered to use ETCO_2_ as a predictor of ROSC [3]. Of note, to reduce the use of animals, the study population came from different experimental series with different CPR strategies. Additionally, acute and chronic illnesses with comorbidities can result in a ventilation/perfusion mismatch, which can limit the accuracy of ETCO_2_ [30] and may be resolved by using PECO_2_ as a marker of ventilation/perfusion mismatch in future studies. Of note, our protocol of giving 100% oxygen after 30 seconds of CC and then administrating epinephrine 60 seconds after commencement of CC and thereafter every minute, is not in line with the current resuscitation guidelines for asphyxiated newborn infants.

## Conclusion

The secondary outcomes of a swine model of neonatal resuscitation demonstrate that continuously monitoring ETCO_2_, VCO_2_, and PECO_2_ during CC has the potential to be a continuous, non-invasive measurement to indicate ROSC, support prediction of outcomes in newborns requiring CC, and determine whether resuscitation efforts should be discontinued. Furthermore, capnometry during CPR can assist in optimizing resuscitation efforts. Further investigation is required to confirm if PECO_2_ is a viable indicator of ventilation/perfusion mismatch for the neonatal population.

## Supporting Information

S1 ARRIVE Checklist (DOC)
